# Granzymes A and K differentially potentiate LPS-induced cytokine response

**DOI:** 10.1038/cddiscovery.2016.84

**Published:** 2016-12-12

**Authors:** Annette C Wensink, Helena M Kok, Jan Meeldijk, Job Fermie, Christopher J Froelich, C Erik Hack, Niels Bovenschen

**Affiliations:** 1 Department of Pathology, University Medical Center Utrecht, Utrecht 3584 CX, The Netherlands; 2 Laboratory of Translational Immunology, University Medical Center Utrecht, Utrecht 3584 CX, The Netherlands; 3 NorthShore University Health Systems Research Institute and University of Chicago, Evanston, IL, USA

## Abstract

Granzymes are serine proteases that, upon release from cytotoxic cells, induce apoptosis in tumor cells and virally infected cells. In addition, a role of granzymes in inflammation is emerging. Recently, we have demonstrated that extracellular granzyme K (GrK) potentiates lipopolysaccharide (LPS)-induced cytokine response from monocytes. GrK interacts with LPS, disaggregates LPS micelles, and stimulates LPS-CD14 binding and Toll-like receptor signaling. Here we show that human GrA also potentiates cytokine responses in human monocytes initiated by LPS or Gram-negative bacteria. Similar to GrK, this effect is independent of GrA catalytic activity. Unlike GrK, however, GrA does not bind to LPS, has little influence on LPS micelle disaggregation, and does not augment LPS-CD14 complex formation. We conclude that GrA and GrK differentially modulate LPS-Toll-like receptor signaling in monocytes, suggesting functional redundancy among cytotoxic lymphocyte proteases in the anti-bacterial innate immune response.

## Introduction

Granzymes are a family of structurally related serine proteases best known for their ability to induce apoptosis in tumor cells or virus-infected cells.^1^ There are five human granzymes: granzyme A (GrA), GrB, GrH, GrK, and GrM. Granzymes are stored in granules of cytotoxic lymphocytes (including cytotoxic T lymphocytes, natural killer (NK) cells, NKT cells, and *γδ* T cells) and are released into the immunological synapse upon recognition of a target cell by a cytotoxic lymphocyte. They subsequently enter the target cell with the aid of the pore-forming protein perforin, and cleave various intracellular substrates that prompt target cells to undergo apoptosis.^2,3^ However, the cytotoxic potential of some granzymes has been debated,^4–6^ and evidence suggests that granzymes fulfill additional extracellular functions. Increased levels of soluble granzymes are found in plasma and synovial fluid of rheumatoid arthritis patients^7,8^ and in serum and broncheoalveolar lavage fluid of patients suffering from bacterial or viral infections.^8–12^ Although the functional consequences of this granzyme release remain incompletely understood, granzymes have been implicated in cytokine release or processing.^5,6,13–17^ This suggests that granzymes are involved in the inflammatory response to infections.

Compared with healthy controls, levels of soluble GrA are elevated in serum from sepsis patients,^7,8,10,18^ and lipopolysaccharide (LPS) injection into healthy volunteers results in GrA release.^11,19^ Increased intracellular GrA levels in cytotoxic lymphocytes correlate with disease severity in sepsis patients,^20^ and GrA^−/−^ mice are more resistant to LPS challenges than WT mice.^6,21^ GrA releases the inflammatory cytokines IL-6, IL-8, IL-1*β* and TNF*α* from human monocytes,^6,22,23^ IL-6 and IL-8 from fibroblasts,^24^ and IL-8 from A549 epithelial cells.^25^ Furthermore, GrA induces human macrophages to produce TNF*α*, which indirectly protects them from mycobacterial infection.^26^ These data suggest that GrA has a role in disease progression of infections and sepsis.

Recently, we have demonstrated that extracellular GrK potentiates LPS-induced release of inflammatory cytokines from monocytes, and that this effect is independent of the catalytic activity of GrK.^13^ GrK binds to LPS and releases single LPS molecules from LPS micelles, thereby lowering the threshold for monocyte activation.^13^ In the present study, we show that GrA alone does not induce cytokine release from human primary monocytes. Similar to GrK, GrA potentiates cytokine responses induced by LPS, independent of its catalytic activity. In contrast to GrK, however, GrA does not bind to LPS, does not efficiently remove LPS molecules from micelles, and does not augment LPS-CD14 complex formation. Apparently, granzymes use different mechanisms to augment LPS-induced cytokine responses from monocytes. This points to functional redundancy among cytotoxic lymphocyte proteases in the anti-bacterial innate immune response.

## Results

### GrA enhances LPS-induced cytokine response from monocytes

**Figure 1:**
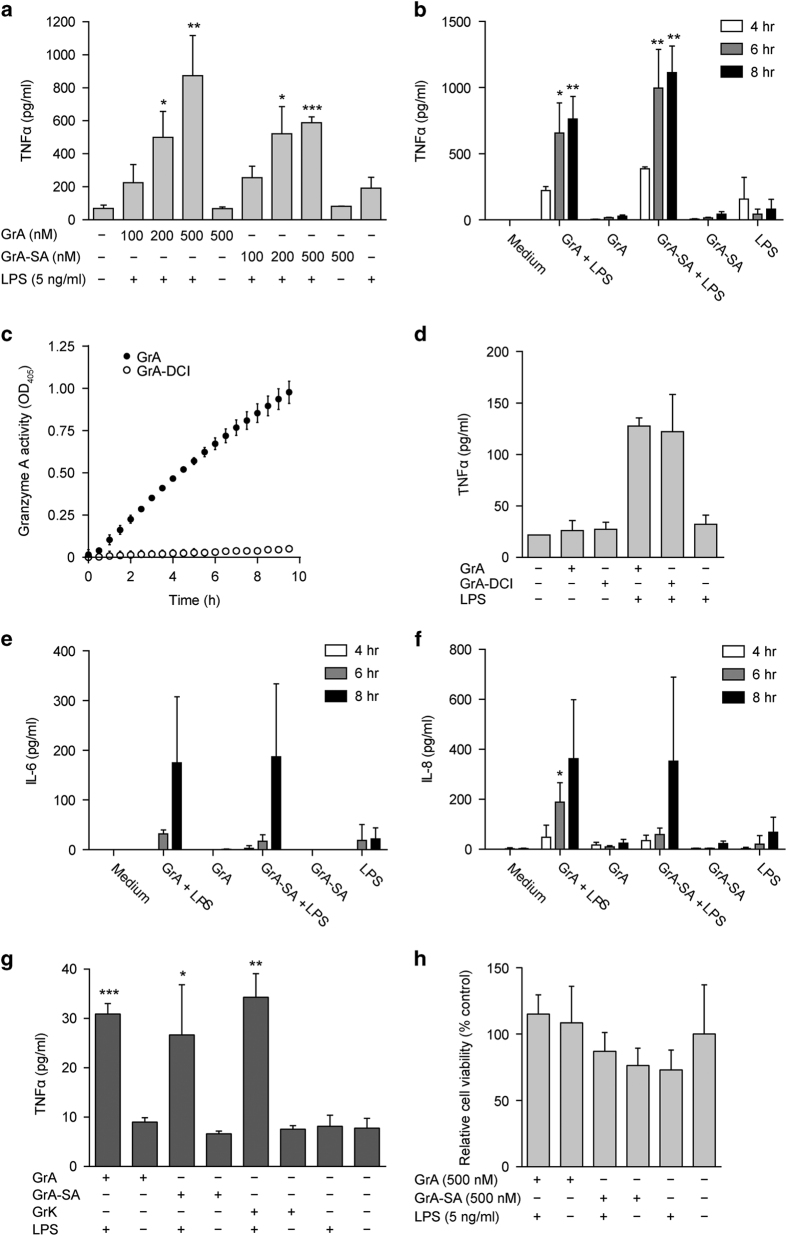
GrA enhances the LPS-induced proinflammatory cytokine release from monocytes. (**a**) Human monocytes were incubated with increasing concentrations of GrA(-SA) with or without LPS (5 ng/ml) for 6 h. Tumor necrosis factor-*α* (TNF*α*) levels in the culture supernatants were determined. Data are expressed as mean±S.D. and are representative of at least three independent experiments with normal donors (*P<0.05; **P<0.01; ***P<0.001, compared with LPS control). TNF*α* release was not statistically different between GrA plus LPS and GrA-SA plus LPS (*P*=0.113). (**b**) Human monocytes were treated with GrA(-SA) (400 nM) with or without LPS (2.5 ng/ml) for 4, 6 or 8 h. TNF*α* was detected in the culture supernatants. Data are expressed as mean±S.D. and are representative of at least three independent experiments with normal donors (*P<0.05; **P<0.01, as compared with LPS control for the same time point). (**c**) GrA (7.5 *μ*M) was treated with DCI (150 *μ*M) for 30 min, dialyzed overnight, and residual GrA activity (2 *μ*M) was kinetically monitored by chromogenic substrate Z-Phe-Arg-pNA (1 mM) hydrolysis at OD405. (**d**) Human monocytes were treated with GrA(-DCI) (500 nM) with or without LPS (1 ng/ml) for 6 h. TNF*α* was detected in the culture supernatants. Data are expressed as mean±range and are representative of three independent experiments in duplo with normal donors. (**e**, **f**) Human monocytes were treated with GrA(-SA) (400 nM) with or without LPS (2.5 ng/ml) for 4, 6 or 8 h. Cytokines interleukin-6 (IL-6) (**e**) and IL-8 (**f**) were detected in the culture supernatants. Data are expressed as mean±S.D. and are representative of at least three independent experiments with normal donors (*P<0.05; **P<0.01, as compared with LPS control for the same time point). (**g**) The magnitude of the GrA synergistic effect is similar to that of GrK. Human monocytes were incubated with GrA(-SA) or GrK (400 nM) with or without LPS (2.5 ng/ml) for 6 h, after which TNF*α* levels in the culture supernatants were measured. Data are expressed as mean±S.D. and are representative of six independent experiments with normal donors (*P<0.05; **P<0.01; ***P<0.001, compared with LPS control). (**h**) GrA is not cytotoxic to human monocytes. Human monocytes were incubated with GrA(-SA) (500 nM) for 0–6 h. Relative cell viability was determined by WST-1 assay. Data (*n*=3 per treatment) are depicted as mean±S.D. (% of medium control) and are representative of at least two independent experiments with normal donors.

**Figure 2:**
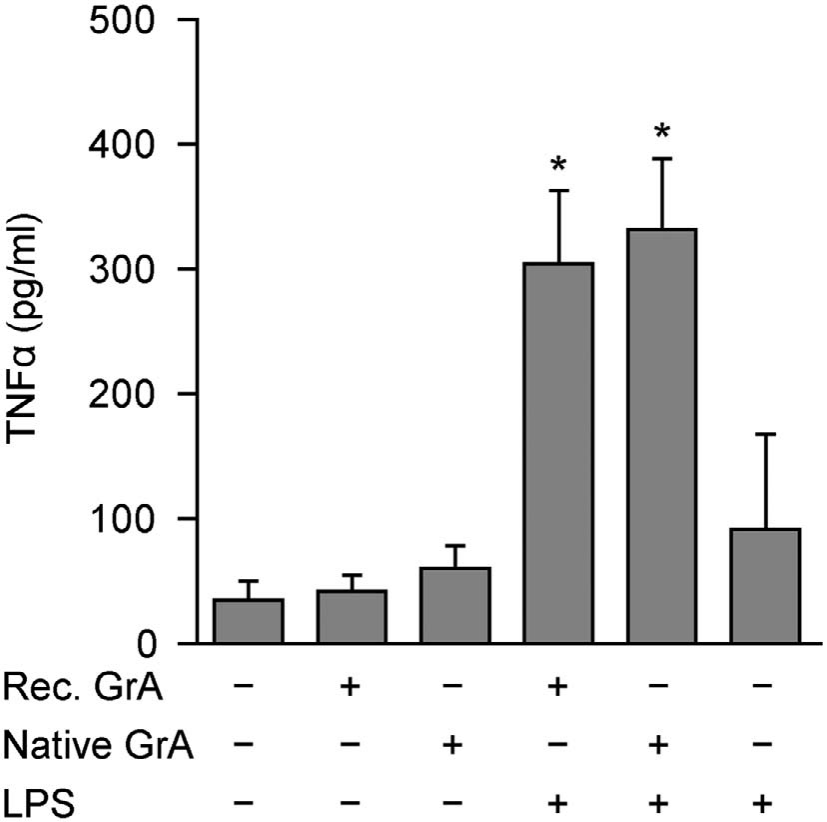
Native human GrA potentiates LPS-induced tumor necrosis factor-*α*(TNF*α*) release from monocytes. Human monocytes were incubated with isolated native human GrA (500 nM) or recombinant GrA (500 nM) with or without LPS (5 ng/ml) for 6 h. TNF*α* levels in the culture supernatants were determined. Data are expressed as mean±S.D. and are representative of at least three independent experiments with normal donors (*P<0.05, compared with LPS control).

**Figure 3:**
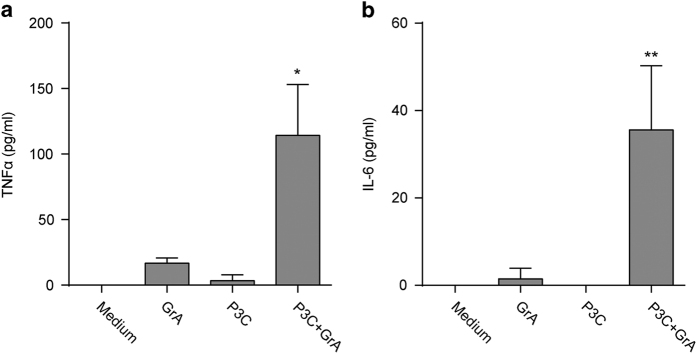
GrA enhances Toll-like receptor 2 (TLR2)-induced proinflammatory cytokine release from monocytes. Human monocytes were incubated with GrA (500 nM) with or without Pam3cys (P3C; 10 ng/ml) for 6 h. Tumor necrosis factor-*α*(TNF*α*) (**a**) and interleukin-6 (IL-6) (**b**) levels in the culture supernatants were determined. Data are expressed as mean±S.D. and are representative of four independent experiments with normal donors (*P<0.05; **P<0.01, compared with P3C only control).

To determine the impact of GrA on LPS-induced cytokine responses, monocytes were incubated with extracellular GrA or its catalytically inactive mutant (GrA-SA) in the presence or absence of a suboptimal stimulatory dose of LPS. Treatment of monocytes with extracellular GrA alone for 0–8 h did not result in cytokine release (Figure 1). However, incubation of monocytes with GrA in combination with a suboptimal stimulatory dose of LPS enhanced TNF*α* release compared with the response to LPS alone in a dose- and time-dependent manner (Figures 1a and b). This effect was independent of GrA catalytic activity, as treatment of monocytes with catalytically inactive GrA-SA showed similar effects as wild-type GrA (Figures 1a and b). In addition, blocking of GrA catalytic activity with the inhibitor 3,4-dichloroisocoumarin (DCI; Figure 1c) did not affect the potency of GrA to stimulate LPS-induced TNF*α* release from monocytes (Figure 1d). IL-6 and IL-8 release were also enhanced in response to combined treatment with GrA and LPS, compared with LPS control (Figures 1e and f). Again, cytokine secretion was independent of GrA catalytic activity, and treatment of monocytes with granzyme alone did not result in substantial IL-6 or IL-8 release (Figures 1e and f). Moreover, GrA had comparable potency to enhance LPS-induced TNF*α* release as GrK (Figure 1g). Treatment of monocytes with GrA alone did not induce cell death (Figure 1h). Native human GrA purified from killer cells behaved similar to recombinant GrA in that native GrA also potentiated LPS-induced TNF*α* response from monocytes (Figure 2). Finally, GrA also potentiated proinflammatory cytokine responses (TNF*α* and IL-6) induced by TLR2 agonist Pam3cys (synthetic triacylated lipopeptide; Figure 3). We conclude that GrA enhances LPS-induced cytokine response in human monocytes, independent of GrA catalytic activity.

### The effect of GrA on LPS-induced cytokine production depends on CD14

**Figure 4:**
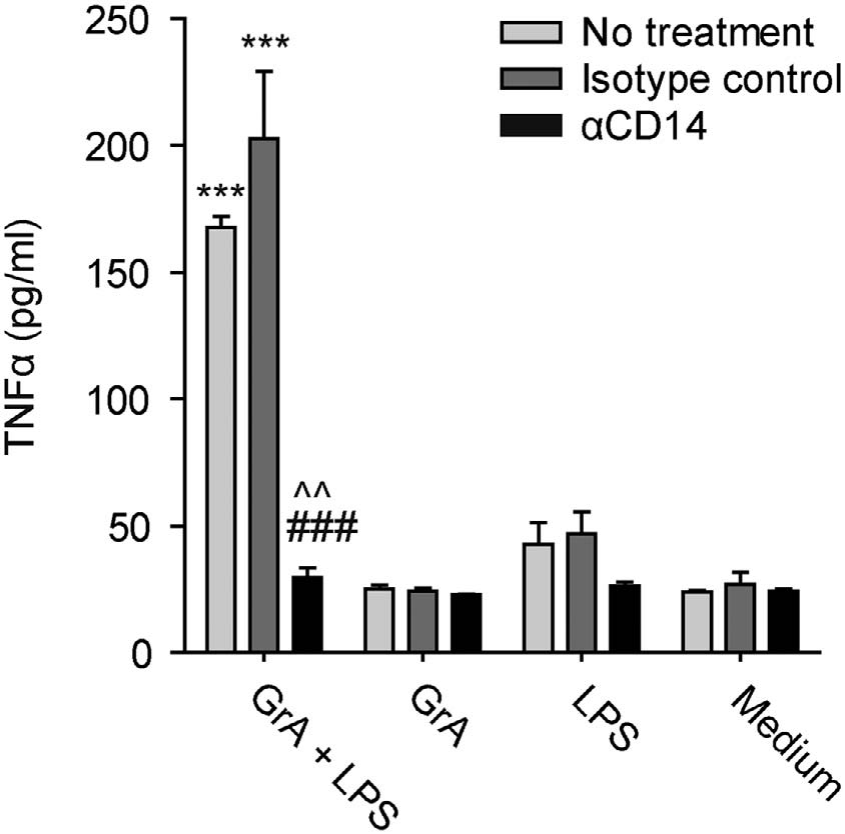
The synergistic effect of GrA on the LPS-induced tumor necrosis factor-*α* (TNF*α*) release is dependent on CD14. Human monocytes were treated with GrA (500 nM) with or without LPS (5 ng/ml) for 6 h in serum-free medium. Cells were pretreated with a neutralizing *α*CD14 antibody, an isotype control or serum-free medium alone (***P<0.001, compared with LPS control; ^###^*P*<0.001, compared with GrA+LPS without antibody pre-treatment; ^^*P*<0.01, compared with GrA+LPS with isotype pre-treatment). Data (*n*=3 per treatment) are depicted as mean±S.D. and are representative of three independent experiments with normal donors.

LPS activates monocytes via binding to CD14 and subsequent transfer to TLRs,^27^ and mice lacking CD14 are highly insensitive to LPS.^28^ If potentiation of LPS-induced cytokine release by GrA is entirely mediated via CD14, neutralization of CD14 would abolish this effect. To examine this, monocytes were preincubated with or without a neutralizing CD14 antibody or isotype control and then treated with GrA with or without LPS. Preincubation with *α*CD14 antibody completely abolished the synergistic effect of GrA and LPS (Figure 4), whereas the isotype control had no effect. Thus, potentiation of LPS-induced cytokine response by GrA is dependent on CD14 signaling.

### GrA enhances TNF*α* release induced by Gram-negative bacteria

**Figure 5:**
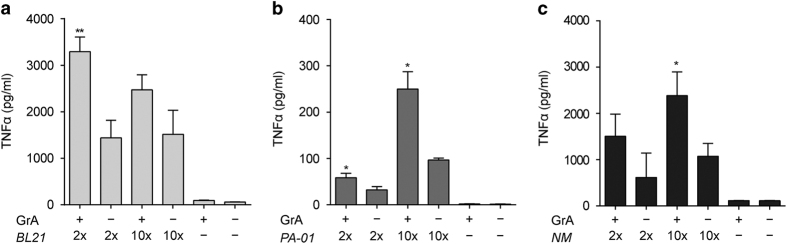
GrA enhances tumor necrosis factor-*α* (TNF*α*) production by human monocytes induced by Gram-negative bacteria. Monocytes were incubated with GrA (500 nM) for 6 h with or without *E. coli BL21* (**a**), *P. aeruginosa* (PA-01) (**b**), or NM *HB-1* (NM) (**c**) at 2- or 10-fold excess compared with cell numbers. Supernatants were analyzed for TNF*α*. Data are depicted as mean±S.D. (*n*=3 per donor) and are representative of at least three independent experiments with normal donors (*P<0.05; **P<0.01, compared with bacteria only).

As LPS is an important constituent of the Gram-negative bacterial cell wall, we wondered whether treatment of monocytes with GrA and live Gram-negative bacteria also potentiates cytokine release. Monocytes were treated with GrA alone, Gram-negative bacteria (*Escherichia coli*, *Pseudomonas aeruginosa*, or *Neisseria meningitides* (NM)) alone, or GrA combined with bacteria. As expected, treatment with only GrA failed to induce cytokine release (Figure 5). Treatment with bacteria resulted in a clear cytokine release, which was significantly enhanced when GrA was added (Figures 5a–c). This demonstrates that GrA augments cytokine release generated by live Gram-negative bacteria.

### GrA binds to Gram-negative bacteria

**Figure 6:**
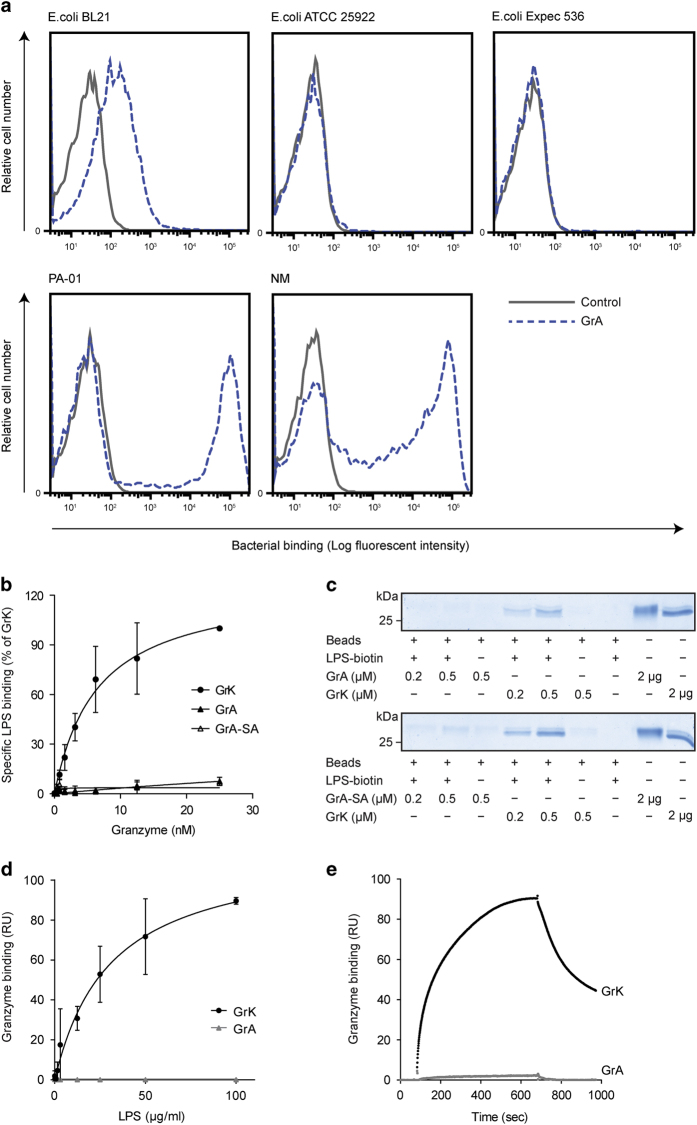
GrA binds several Gram-negative bacteria but not LPS. (**a**) GrA binds to Gram-negative bacteria. GrA-biotin binding to *E. coli BL21*, *E. coli ATCC 25922*, *E. coli Expec 536*, *P. aeruginosa* (PA-01), or NM *HB-1* (NM) was detected by flow cytometry. Data are representative of at least three independent experiments. (**b**) GrA does not bind to LPS in a solid-phase binding assay. LPS was immobilized and incubated with biotinylated GrK, GrA, or GrA-SA. Data are depicted as specific binding (depicted as % of maximum GrK binding) and represent mean±S.D. of three independent experiments. (**c**) GrA does not bind to LPS in a pull-down assay. LPS-biotin was coupled to streptavidin-sepharose beads. After washing, beads were incubated with GrA (upper panel) or GrA-SA (lower panel). GrK was used as a positive control. Bound protein was eluted from the beads and visualized by sodium dodecyl sulfate-polyacrylamide gel electrophoresis (SDS-PAGE) followed by total protein staining. Results are representative for three independent experiments. (**d**) GrA does not bind to LPS in a surface plasmon resonance assay. Immobilized GrA and GrK were incubated with LPS for 10 min. (association), followed by a buffer flow (dissociation). The association of LPS to GrA and GrK at *t*=10 min is shown. Results represent mean±S.D. of three independent experiments. RU, response units. (**e**) Direct comparison of LPS (100 *μ*g/ml) binding with immobilized GrK and GrA. Results are representative of three independent experiments. RU, response units.

GrK binds to several Gram-negative bacteria.^13^ To determine whether GrA also binds to Gram-negative bacteria, several bacteria were incubated with or without biotinylated GrA and binding was measured by flow cytometry. GrA bound to *E. coli BL21*, *Pseudomonas aeruginosa* (PA-01), and NM (Figure 6a), with a relative binding efficiency of NM>PA-01>*E. coli BL21*. These same Gram-negative bacteria also bind to GrK with differential efficiency (*E. coli* *BL21*>NM>PA-01).^13^ Similar to GrK,^13^ GrA did not bind to *E. coli ATCC 25922* or *E. coli Expec 536* (Figure 6a). For some bacterial strains (PA-01 and NM), two distinct populations of bacteria were observed, one that bound GrA and one that did not. This possibly reflects heterogeneity among these bacteria. These data indicate that GrA binds to some Gram-negative bacteria.

### GrA does not bind to LPS

We have shown that GrK binds to LPS, and that this binding contributes to the synergistic effect of GrK on the LPS-induced cytokine release from monocytes.^13^ Considering that GrA binds to Gram-negative bacteria and also enhances the LPS-induced cytokine response in monocytes, we investigated whether this granzyme also binds to LPS. First, biotinylated GrA(-SA) was incubated on immobilized LPS in a solid-phase binding assay and specific binding was determined, using GrK as a positive control. In marked contrast with GrK, GrA and GrA-SA did not bind to LPS (Figure 6b). Second, a pull-down assay was performed in which biotinylated LPS was coupled to streptavidin-coated beads that were incubated with GrA, GrA-SA, or GrK as a positive control. Bound protein was analyzed by SDS-PAGE. In contrast to GrK, which efficiently bound LPS, binding of GrA or GrA-SA to LPS-coated beads was barely observed (Figure 6c). Finally, surface plasmon resonance experiments were performed, using immobilized GrA and GrK to which LPS was applied. Whereas LPS bound to GrK in a dose-dependent manner (Figure 6d), binding of LPS to immobilized GrA was hardly detectable even at the highest LPS concentration used (Figure 6e). In summary, these data show that GrA does not bind to LPS.

### GrA inefficiently removes individual LPS molecules from micelles and does not augment LPS-CD14 complex formation

**Figure 7:**
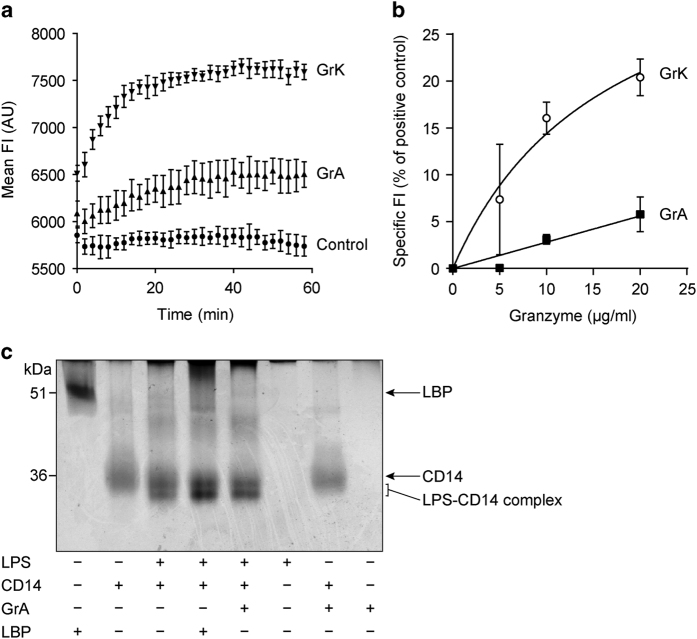
GrA does not efficiently remove LPS from micelles and does not augment LPS-CD14 complex formation. (**a**) LPS-BODIPY-FL-FL, of which the fluorescent intensity (FI) increases upon removal from LPS micelles, was incubated for 90 min at 37 °C, after which granzymes (20 *μ*g/ml) or extra PBS (LPS-BODIPY-FL control) were added. The FI was then measured for an additional 60 min. Results are depicted as mean±S.D. (*n*=3) and are representative of two independent experiments. Explanation of legends in figure: GrA=LPS-BODIPY-FL with GrA; GrK=LPS-BODIPY-FL with GrK, Control+LPS-BODIPY-FL alone. (**b**) LPS-BODIPY-FL was incubated with GrA or GrK and the mean FI was measured. Data are corrected for the FI of LPS-BODIPY-FL alone and depicted as the percentage of the FI of LPS-BODIPY-FL treated with 2% SDS. Data represent mean±S.D. (*n*=6). (**c**) LPS (2.5 *μ*g) was incubated with human recombinant CD14 (0.5 *μ*g) with or without LBP (0.5 *μ*g) or GrA (0.5 *μ*g) for 2 h at 37 °C. LPS-CD14 complex formation was analyzed by a band shift on native polyacrylamide gel electrophoresis (PAGE) followed by silver staining. Band intensities were quantified and showed that GrA did not augment LPS-CD14 complex formation.

LPS is an amphipathic molecule, forming micelles in aqueous solutions. We have previously demonstrated that GrK liberates individual LPS molecules from these micelles, thereby augmenting LPS-CD14 complex formation.^13^ To study the behavior of GrA in this respect, we incubated GrA with LPS-BODIPY-FL. The fluorescence of this compound is quenched when LPS is in micelles and increases upon removal from the micelle. When GrK (positive control) was added to LPS-BODIPY-FL, a clear increase in FI (fluorescent intensity) was observed (Figure 7a). An increase in FI was also observed when GrA was added, but ~4-fold less efficient as compared with GrK. The FI of LPS-BODIPY-FL alone remained constant during measurements (Figure 7a). Experiments with increasing concentrations of GrA and GrK showed similar results (Figure 7b). Next, we wondered whether this limited effect of GrA would free a sufficient amount of single LPS molecules to augment LPS-CD14 complex formation, which lowers the threshold for monocyte activation by LPS.^27,29^ LPS and recombinant CD14 were incubated in the absence or presence of GrA or (positive control) LBP. LPS-CD14 complex formation was visualized by native PAGE and total protein stain (silver stain), as described previously.^29^ While LBP stimulated complex formation between LPS and CD14, this was virtually not the case for GrA (Figure 7c). We conclude that GrA can disaggregate LPS micelles to some extent, but does not augment LPS-CD14 complex formation.

## Discussion

Growing evidence points to a role for granzymes in infection and inflammation.^1,30^ Levels of soluble granzymes are increased in the circulation during inflammation and contribute to cytokine release and processing.^6,13,15,16,31,32^ GrA may be important in the disease progression of sepsis,^11,20^ but its extracellular functions are incompletely understood. Here we demonstrate that GrA augments LPS-induced cytokine response in human monocytes (Figures 1 and 2), in a CD14-dependent manner (Figure 4). GrA, similar to GrK,^13^ did not enhance IFN*β* release (data not shown), suggesting that the granzyme mainly affects cytokine release via the MyD88 pathway. Unlike GrK, however, GrA does not bind to LPS (Figure 6), does not efficiently remove LPS from micelles, and does not enhance LPS-CD14 complex formation (Figure 7). Therefore, GrA augments LPS-induced cytokine response from human monocytes via mechanisms at least partially different from those used by GrK.^13^

Treatment of monocytes with GrA alone did not induce cytokine production (Figures 1, 2, 3, 4, and 5). These results are in accordance with our previous data obtained with GrK,^13^ but stand in contrast to earlier results showing that low doses of GrA (~50–200 nM) induce production of the cytokines TNF*α*, IL-1*β*, IL-6, and IL-8 in human primary monocytes.^6^ This effect is dependent on GrA catalytic activity and is enhanced upon intracellular GrA delivery.^6^ We here show that cytokine production by GrA requires costimulation with low doses of LPS (Figure 1) or Gram-negative bacteria (Figure 5), and is not dependent on GrA catalytic activity (Figure 1). We have no clear explanation for the discrepancy between our results and those published in earlier work,^6^ other than that monocyte differentiation status or monocyte cell numbers, may have been different in the experimental conditions. Mouse GrA^6^ and mouse GrK^5^ induce IL-1*β* release in mouse macrophages that have been sensitized with LPS before the experiment. Furthermore, GrA^−/−^ and GrM^−/−^ mice survive longer than WT mice when challenged with LPS,^6,17^ and GrM^−/−^ mice produce less cytokine upon LPS injection, compared with WT mice.^17^ These results indicate that granzymes enhance the innate immune response to LPS, at least via potentiating LPS-induced cytokine responses.^13^ It remains unclear whether or not perforin-mediated intracellular delivery of granzymes is required for these effects of granzymes.^5,6,17,21^

What is the role and source of extracellular GrA during bacterial sepsis? Arias *et al*.^21^ recently have used a mouse model of infection with the Gram-negative bacterial pathogen *Brucella microti* to analyze the capacity of killer cell subsets to control bacterial infection and sepsis.^21^ When injected with a sublethal dose of this pathogen, wild-type and GrA^−/−^ mice are able to clear the infection, whereas bacteria survive in mice knockout for perforin or GrB, as well as in mice depleted of cytotoxic T cells.^21^ Interestingly, following a fatal challenge, only GrA^−/−^ mice show increased survival, which correlated with reduced levels of proinflammatory cytokines in the blood. In this experimental setup, GrA was derived from NK cells, as transfer of wild-type, but not GrA^−/−^, NK cells into GrA-deficient recipient mice restores the susceptibility to sepsis.^21^ This shows that proinflammatory cytokine induction and infection-related pathology, but not bacterial clearance, requires GrA. These data are compatible with our data that GrA potentiates LPS-induced proinflammatory cytokine response. Spencer *et al.*^26^ report that GrA released by *γδ* T cells induces production of TNF*α* in human macrophages infected with mycobacteria. This TNF*α* production, in turn, inhibits growth of the intracellular mycobacteria.^26^ The authors show that TNF*α* produced by the macrophages, and not by the *γδ* T cells, is responsible for this effect.^26^ It has been reported that mycobacteria activate infected macrophages to produce TNF*α* via TLR2.^33^ Furthermore, infected monocytes and macrophages frequently undergo apoptosis.^34,35^ Thus, it is feasible that GrA enhances TNF*α* production induced by mycobacterial products, released from infected or apoptotic macrophages. This opens the possibility that granzymes also enhance immune responses to TLR ligands other than LPS.

Previously, we have demonstrated that GrK binds to LPS and to several Gram-negative bacteria.^13^ We hypothesized that GrK binding to Gram-negative bacteria is mediated via their LPS moieties and that differences in LPS structure between different bacterial strains could explain differences in GrK binding intensity. In the present study, we found that GrA binds the same Gram-negative bacteria as GrK (Figure 6),^13^ whereas GrA does not bind to LPS (*E. coli* B111:O4; Figure 6). Whether granzyme binding to Gram-negative bacteria depends on the LPS subtype or is driven by other bacterial molecules remains an open question that deserves further study.

As GrA does not bind to LPS, and does not efficiently liberate LPS molecules from micelles, other mechanisms likely contribute to the synergistic effect of GrA on LPS-induced cytokine production. Azurocidin (an inactive serine protease structurally related to granzymes) also enhances the LPS-induced cytokine release from human monocytes^36^ and internalization is a prerequisite for this effect.^37,38^ Furthermore, GrA binds to and is internalized by monocytes.^6^ This opens the possibility that GrA influences LPS signaling intracellularly in a perforin-independent manner. Intracellular delivery of GrA and GrK by perforin or perforin analogs shows beneficial effects on granzyme-induced cytokine responses.^5,6^ One mechanism explaining this effect has recently been elucidated by Hildebrand *et al*.^23^ who show that GrA produces bioactive IL-1*β* via a nonapoptotic, inflammasome- and caspase-1-independent pathway. However, this mechanism relies on GrA catalytic activity, and contrasts with our data that GrA potentiates LPS-induced cytokine response independent of GrA catalytic activity. Additional research is required to further identify the molecular mechanism(s) by which GrA enhances cytokine production independent of its catalytic activity.

In conclusion, GrA and GrK^13^ use differential mechanisms to enhance TLR4 signaling during bacterial infections. Apparently, granzymes augment inflammation in manners sufficiently different from each other to provide back-up mechanisms. This ensures a proper innate immune response when one or more granzymes are blocked. The possibility of functional redundancy further underlines the potential importance of cytotoxic lymphocyte proteases in augmenting the anti-bacterial innate immune response.

## Materials and methods

### Reagents

Cell culture reagents were from Gibco (Thermo Scientific, Waltham, MA, USA), unless stated otherwise. Cell proliferation reagent (WST-1 reagent) was from Roche Applied Science (Penzberg, Germany). Human AB serum was from Invitrogen (Thermo Scientific). All yeast culture compounds were from Becton, Dickinson and Company (Erembodegem, Belgium). Synthetic chromogen substrats Z-Phe-Arg-pNA for GrA and Ac-Lys-pNA were from Bachem (Bubendorf, Switzerland). LPS (*E. coli* 0111:B4) and LPS-BODIPY-FL were from Sigma-Aldrich (Zwijndrecht, The Netherlands). Biotin-conjugated LPS (*E. coli* 0111:B4) was from Invivogen (Toulouse, France). Recombinant CD14 and LBP were from R&D Systems (Abingdon, UK). Pam3cys was from Invivogen. Polyclonal antibody to human nucleosome assembly protein SET was from Alexis Biochemicals (Enzo Life Sciences, Farmingdale, NY, USA). DCI was from Sigma-Aldrich. Monoclonal antibody to human CD14 was from R&D Systems. IgG1 isotype control used in CD14-neutralizing experiments was anti-human serpin B13 antibody (clone 4A9D).^39^ Secondary antibodies were obtained from Jackson Immunoresearch (Suffolk, UK). All bacterial strains were kind gifts from the Department of Medical Microbiology (UMC Utrecht, The Netherlands).

### SDS-PAGE

Proteins were separated on a 10 or 12% SDS-PAGE gel, and total protein staining was performed with Instant Blue (Expedeon Ltd, Swavesey, UK).

### Production, purification and characterization of granzymes

Human GrA and GrK, and the GrA catalytically inactive mutant (GrA-SA), in which the active site residue Ser195 has been replaced by Ala, were produced and characterized as described before.^40,41^ Briefly, cDNA encoding GrA or GrK was cloned into the yeast expression vector pPIC9 (Invitrogen, Thermo Scientific). The catalytically inactive GrA-SA mutant was generated using QuikChange Site-Directed Mutagenesis Kit (Stratagene, Agilent, Santa Clara, CA, USA) according to the manufacturer’s protocol. Plasmids were transformed into the GS115 strain of *Pichia pastoris* (Invitrogen, Thermo Scientific) and granzymes were expressed in conditioned media for 72 h. Granzymes were purified by cation exchange chromatography followed by affinity chromatography. Purified granzymes were dialyzed against 20 mM Tris, 150 mM NaCl, pH 7.4. Alternatively, granzymes were dialyzed against 1x PBS for use in surface plasmon resonance experiments or for biotinylation (see below). Protein concentrations were measured using Bradford (Bio-Rad, Hercules, CA, USA) or Nanodrop (Thermo Scientific) and granzymes were stored at −80 °C until use. All active granzymes cleaved their respective synthetic chromogenic substrates (Ac-Lys-pNA for GrK and Z-Phe-Arg-pNA for GrA), whereas inactive GrA-SA did not cleave Z-Phe-Arg-pNA (data not shown). In addition, GrA and GrK both cleaved their known macromolecular substrate SET (data not shown).^40,42^ This indicates that GrA and GrK are catalytically active, whereas GrA-SA is not. Granzyme batches were not contaminated with endotoxin (<1.5 EU/ml (~0.15 ng/ml), final concentration) as determined by LAL assay (Thermo Scientific) on GrK and GrA-SA preps. GrA batches could not be tested, as GrA directly hydrolyzes the substrate (IGAR-pNA) used in the LAL assay. Granzymes were biotinylated using the Biotin Protein Labeling Kit (Roche Applied Science) according to the manufacturer’s protocol. To block GrA catalytic activity, GrA (7.5 *μ*M) was treated with DCI (150 *μ*M) for 30 min at RT, and GrA-DCI was subsequently dialyzed against 20 mM Tris, 150 mM NaCl, pH 7.4. GrA-DCI inactivation was confirmed. Human native GrA was isolated from IL-2-activated lymphocytes and endotoxin was removed with the EndoTrap-Blue Kit (Cambrex Biosciences, East Rutherford, NJ, USA) as described previously.^6^ We have never observed any cell death induced by incubation of human monocytes with up to 1 *μ*M of GrA or GrA-SA alone.

### Solid-phase binding assays with granzymes and LPS

LPS (10 *μ*g/ml in PBS) was incubated overnight at 4 °C on 96-well plates (Greiner Bio-One GmbH, Kremsmünster, Austria), and incubated with various concentrations of biotinylated GrA(-SA) in PBS with 0.1% (v/v) Tween-20 at 37 °C for 2 h. GrK was used as a positive control. Bound granzymes were visualized by incubation with Streptavidin-polyHRP (Sanquin, Amsterdam, The Netherlands), followed by TMB (Invitrogen, Thermo Scientific). The reaction was stopped by adding 1 M H_2_SO_4_ and OD450 was measured.

### LPS-Granzyme pull-down assay

LPS-biotin (50 *μ*g/ml) was coupled to streptavidin-coated beads (Amersham Biosciences, GE Healthcare, Chicago, IL, USA). After extensive washing, the beads were incubated with recombinant GrA(-SA) or GrK for 1 h at RT or overnight at 4 °C by head over head rotation. Bound protein was eluted from the beads with 2x concentrated Laemmli buffer, and analyzed by SDS-PAGE followed by Instant Blue total protein staining.

### Surface plasmon resonance analysis

Real-time binding experiments were performed on the Biacore T100 (GE Healthcare). Granzymes were immobilized on CM5 sensor-chip surface via amine coupling at 3001 (GrA) and 2866 (GrK) response units, using the manufacturer’s instructions. One control flow channel was routinely activated and blocked in the absence of protein. Association of LPS (0–100 *μ*g/ml) was assessed in triplicate in PBS for 10 min, at a flow rate of 5 *μ*l/min at 37 °C. Dissociation was allowed for 5 min in the same buffer flow. Sensor chips were regenerated using several pulses of 50 mM Tris (pH 7.4) and 1 M NaCl at a flow rate of 20 *μ*l/min. Data were corrected for aspecific binding of LPS to the control channel, which was <10% of specific binding.

### Binding of GrA to bacteria

Bacteria were diluted in PBS to OD ~0.5 (660 nm), spun down, resuspended in PBS supplemented with 0.1% (w/v) BSA and mixed with biotinylated granzyme (0–20 *μ*g/ml). The mixtures were incubated for 1 h at 37 °C. Bacteria were washed two times with PBS supplemented with 1% BSA, and incubated with 1 *μ*g/ml streptavidin-PE (Southern Biotech, Birmingham, AL, USA) in PBS supplemented with 1% BSA for 60 min at 4 °C. Bacteria were washed once with PBS with 1% BSA and analyzed by flow cytometry. Neither biotin nor streptavidin binds to *E. coli*, *Pseudomonas aeruginosa*, or NM, as demonstrated previously.^43,44^

### Mononuclear cell isolation

Peripheral blood mononuclear cells (PBMCs) were obtained from human donor blood. Briefly, Ficoll-Paque (GE Healthcare) density centrifugation was used to separate fresh blood from healthy volunteers into layers. The mononuclear cell fraction was collected and washed three times with RPMI-1640 containing 5% (v/v) fetal calf serum (FCS; Gibco, Thermo Scientific) and 0.2% bicarbonate (w/v). Cells (0.5×10^6^ cells per well) were incubated for 2 h in 48-well culture plates in RPMI-1640 containing 5% (v/v) AB serum, 2 mM l-glutamine, bicarbonate, and penicillin and streptomycin (P/S), after which non-adherent cells were removed. Adherent cells were subsequently cultured in RPMI-1640 supplemented with 2% AB serum and 0.2% bicarbonate for up to 1 week before use. Alternatively, monocytes were purified from the PBMC layer using magnetic-activated cell sorting (MACS). The PBMC layer was washed once with RPMI-1640 containing 5% FCS and bicarbonate and once with ice-cold PBS containing 0.5% FCS and 2 mM EDTA. Monocytes were subsequently isolated using a CD14 MACS Kit (Miltenyi Biotec, Bergisch Gladbach, Germany). The positive fraction was washed once with RPMI-1640 containing 5% human AB serum, 2 mM l-glutamine, 0.2% bicarbonate, and P/S, and once with serum-free RPMI containing 0.2% bicarbonate.

### Proinflammatory cytokine response in isolated human monocytes

Monocytes (0.5×10^5^/well) were incubated with granzyme (0–500 nM) with or without LPS (0–5 ng/ml) or Pam3cys (10 ng/ml) in serum-free medium for 0–8 h depending on the experiment. After incubation, supernatants were collected and stored at −20 °C. Cells were subjected to a WST-1 assay to determine relative cell viability. To each well, 250 *μ*l WST-1 reagent was added and the increase in OD450 was measured for 90 min. Wells without cells were used as a negative control. Experiments with bacteria, GrA(-SA), and monocytes were carried out in the same way, except that LPS was replaced with bacteria, added in a 2–10-fold excess compared with the number of monocytes per well. The effect of *α*CD14 mAb was tested in a similar experiment, except that monocytes were pretreated for 30 min at 37 °C with *α*CD14 mAb (10 μg/ml, which was sufficient to fully block LPS-induced TNF*α* release from monocytes at LPS concentrations of at least 5–100 ng/ml, isotype control (anti-human serpin B13 antibody, 10 *μ*g/ml), or serum-free medium alone. TNF*α*, IL-1*β*, IL-6, and IL-8 levels in culture supernatants were measured using a multiplex assay as described previously^45^ on a Luminex FlexMap 3D (Bio-Rad) with the xPonent 4.2 software (Luminex, Austin, TX, USA). Data were analyzed using BioPlex Manager 6.1.1 (Bio-Rad). Alternatively, TNF*α* was measured using ELISA (PeliKine human TNF*α* ELISA Kit; (Sanquin)).

### Effect of GrA on LPS micelle formation

LPS micelle formation was studied using LPS-BODIPY-FL as described.^36^ The fluorescence of LPS-BODIPY-FL increases upon disaggregation of LPS micelles. Increasing concentrations of granzyme (0–20 *μ*g/ml) were added to LPS-BODIPY-FL (7.5 *μ*g/ml) in 250 *μ*l PBS. LPS-BODIPY-FL alone was used as a negative control and LPS-BODIPY-FL plus GrK served as a positive control. Disaggregation of LPS micelles upon treatment of LPS-BODIPY-FL with 2% SDS was set at 100%. Fluorescent intensity of all samples was measured kinetically for 2 h at 37 °C at 520 nm using the FluoStar Omega apparatus (BMG Labtech, Ortenberg, Germany).

### LPS-CD14 complex formation

LPS (2.5 *μ*g) was incubated with human recombinant CD14 (0.5 *μ*g) with or without LBP (0.5 *μ*g) or GrA (0.5 *μ*g) in 20 mM Tris, 150 mM NaCl, pH 7.4, for 2 h at 37 °C. LPS-CD14 complex formation was analyzed by a band shift on native PAGE followed by silver staining, as described previously.^29^

### Statistical analysis

Unless indicated otherwise, data are depicted as mean values±S.D. and statistical analyses were performed using the independent-samples *t*-test. Two-tailed *P*-values below 0.05 were considered statistically significant.
